# Lipocalin 2—A bone‐derived anorexigenic and β‐cell promoting signal: From mice to humans

**DOI:** 10.1111/1753-0407.13504

**Published:** 2023-11-30

**Authors:** Yuying Yang, Jianmin Liu, Stavroula Kousteni

**Affiliations:** ^1^ Department of Endocrine and Metabolic Diseases, Rui‐jin Hospital, Shanghai Jiao Tong University School of Medicine Shanghai Institute of Endocrine and Metabolic Diseases, and Shanghai Clinical Center for Endocrine and Metabolic Diseases Shanghai China; ^2^ Key Laboratory for Endocrine and Metabolic Diseases of the National Health Commission of the PR China, Shanghai National Clinical Research Center for Metabolic Diseases, Shanghai National Center for Translational Medicine, Rui‐jin Hospital Shanghai Jiao Tong University School of Medicine Shanghai China; ^3^ Department of Physiology and Cellular Biophysics Columbia University Medical Center New York New York USA

**Keywords:** appetite, energy metabolism, Lipocalin‐2, MC4R, β‐cell

## Abstract

The skeleton is traditionally known for its structural support, organ protection, movement, and maintenance of mineral homeostasis. Over the last 10 years, bone has emerged as an endocrine organ with diverse physiological functions. The two key molecules in this context are fibroblast growth factor 23 (FGF23), secreted by osteocytes, and osteocalcin, a hormone produced by osteoblasts. FGF23 affects mineral homeostasis through its actions on the kidneys, and osteocalcin has beneficial effects in improving glucose homeostasis, muscle function, brain development, cognition, and male fertility. In addition, another osteoblast‐derived hormone, lipocalin 2 (LCN2) has emerged into the researchers' field of vision. In this review, we mainly focus on LCN2's role in appetite regulation and glucose metabolism and also briefly introduce its effects in other pathophysiological conditions, such as nonalcoholic fatty liver disease, sarcopenic obesity, and cancer‐induced cachexia.

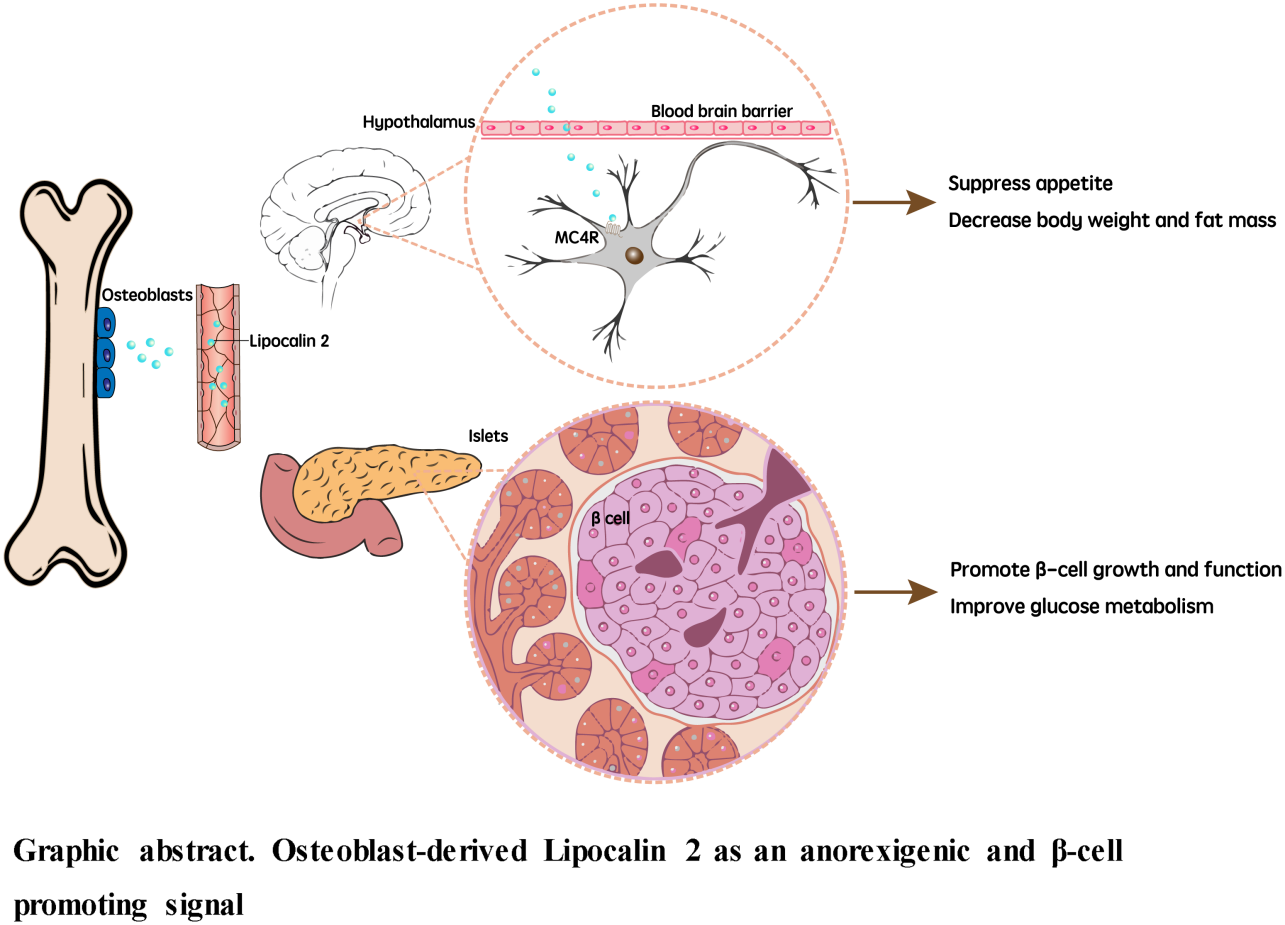

## Graphical abstract

- Lipocalin 2 (LCN2) suppresses appetite through melanocortin‐4 receptor.
- LCN2 protects β‐cell from damage and enhance their proliferation.
- LCN2 orchestrates energy metabolism by maintaining homeostasis of lipid metabolism in liver and supporting muscle regeneration.
- Targeting LCN2 holds significant therapeutic potential for managing both metabolic disorders and cachexia.

## 1 LIPOCALIN 2 AS A SATIETY SIGNAL

Bone is an endocrine organ secreting several bone‐derived molecules and fulfilling its diverse functionalities beyond bone.^1^, ^2^, ^3^, ^4^, ^5^, ^6^, ^7^, ^8^, ^9^ Lipocalin 2 (LCN2) is a 25 kDa secreted glycoprotein belonging to the lipocalin family.^10^, ^11^ It is upregulated in response to bacterial infection and tissue injury^10^, ^12^ and was regarded as an inflammatory cytokine. Its classification in the same family as retinol binding protein 4 had led to the inference that LCN2 is an adipokine.^13^ However, it has been demonstrated that osteoblasts, rather than adipocytes, are the primary source of LCN2. The expression level of LCN2 in osteoblasts is at least 10‐fold higher than that in white adipocytes, and nearly two thirds of circulating LCN2 is derived from osteoblasts.^14^

In recent years, the metabolic function of LCN2 as an anorexigenic signal has been identified in mice, monkeys, and humans.^14^, ^15^ It was revealed that LCN2 levels in the circulation increase by threefold in mice fed after overnight fasting, suggesting its involvement in appetite regulation.^14^ This response is contributed specifically by osteoblasts. To further investigate whether LCN2 is involved in suppression of food intake, osteoblast‐specific LCN2‐deficient mice (LCN2^osb−/−^) were generated. Compared to wild‐type mice, LCN2^osb−/−^ mice exhibited hyperphagia and continuous weight gain. However, when LCN2 was administered to LCN2^osb−/−^ mice, appetite suppression was restored, and their body weight became comparable to that of the control mice.^14^ These findings indicate that LCN2 suppresses appetite and at the same time regulates postprandial food intake.

To determine the translational potential of LCN2 in humans, serum LCN2 levels in lean and obese women were measured after a meal at different time points. Serum LCN2 levels increased in healthy lean women and reached their peak at around 60 min postprandially.^15^ However, this increase was not observed in overweight and obese women. LCN2 serum concentration was even decreased in these subjects after a meal, although they had a higher level at baseline. Phenotypically, compared to the “responsive” group who exhibited higher LCN2 levels after a meal, women in the “nonresponsive” group, defined as a decrease in postprandial LCN2 levels at all time points examined, had higher body mass index (BMI), waist‐to‐hip ratios, diastolic blood pressure, and fasting glucose level. The inverse relationship between LCN2 levels and hunger score observed in the responsive group was also completely lost in the non‐responsive group.^15^ All these results suggest that the postprandial increase in LCN2 levels in lean subjects as a satiety signal is impaired in individuals with obesity or severe overweight.^15^ This observation also echoes the results from a previous human study, showing the dramatic increase of postprandial circulating LCN2 levels in normal weight women after high‐fat meal but not in obese subjects, even though the serum LCN2 concentration in these obese women at baseline are higher than that in normal weight women.^16^

Other findings from mice and human studies provided indirect clues suggesting the effects of LCN2 on energy metabolism. Serum LCN2 concentrations are correlated positively with age in humans.^17^, ^18^ In addition, LCN2 expression in bone is higher in mice during mechanical unloading and in the serum of healthy subjects undergoing 2 weeks of bed rest,^19^, ^20^, ^21^ suggesting a role of LCN2 as a mechanoresponsive gene involved in bone metabolism.^17^, ^18^ It is possible that because energy expenditure during mechanical unloading or at rest in the elderly is decreased, LCN2 levels are increased as part of a homeostatic mechanism to suppress appetite and maintain healthy body weight. However, this hypothesis should be further investigated.

## 2 ROLE OF LCN2 IN INSULIN RESISTANCE AND Β‐CELL FUNCTION

It was intriguing to find that, seemingly paradoxically, in obese and diabetic mice, as well as in mice fed a high‐fat diet (HFD), the levels of serum concentrations of LCN2 are significantly elevated.^22^ Similarly, in humans, the larger the BMI, waist circumference and fat percentage, the higher the circulating LCN2 concentrations.^23^, ^24^ Thus, because LCN2 acts as an anorexigenic signal, the next step was to examine whether LCN2 elevation in obese mice and humans might be a compensatory mechanism to combat obesity, insulin resistance, and other metabolic dysregulations.

Several observations support this hypothesis. First, in prediabetes women, especially in overweight subjects with BMI >30 and 35 kg/m^2^, LCN2 concentration is positively related with BMI, body weight, and homeostatic model assessment for insulin resistance (HOMA‐IR).^24^ Yet, baseline LCN2 levels could not predict the risk of type 2 diabetes in postmenopausal prediabetes women who were followed up for 5 years.^24^ These findings suggested that the increase of LCN2 in prediabetes is not causative for diabetes but rather a response to prediabetes. Second, serum LCN2 levels were upregulated in four different mouse models of obesity (Lepr^ob/ob^, Lepr^db/db^, Mc4r^−/−^, and HFD‐fed mice) as compared to wide‐type littermate controls. Further experiments in HFD‐mice confirmed that the elevated circulating LCN2 was mainly derived from bone, rather than adipose tissue, kidney, or liver. Third, transgenic mice overexpressing *Lcn2* in osteoblasts displayed less food intake, reduced fat mass, body weight, and lower fed and fasting glucose level; more insulin sensitivity; and greater energy expenditure throughout life indicates that prolonged exposure to increased levels of LCN2 does not lead to obesity and also does not cause resistance to the beneficial effects of LCN2.^24^ Taken together these findings suggest that the increased levels of LCN2 may exert protective role in obesity.

Indeed, LCN2 silencing in obese db/db mice further increased food intake and worsened their metabolic phenotype. β‐cell mass and function were compromised and at the same time insulin resistance and body weight gain were increased.^14^ Another study also observed decreased insulin level in LCN2^−/−^ mice.^25^ Conversely, LCN2 treatment suppressed appetite, improved glucose tolerance and insulin resistance, and decreased body weight in lean and obese mice.^24^ This effect is not only just the consequence of the appetite‐suppressing and weight‐reducing capacity of LCN2 but also due to its direct action on β‐cells as LCN2 protect β‐cell from damage and enhance their proliferation.^24^ In agreement with these observations, in a group of diabetes patients with similar BMI and age as prediabetes cohort in the same study, serum LCN2 level is negatively correlated with HbA1c concentration, and positively related with improved β‐cell function (HOMA‐B).^24^ In addition, LCN2 can stimulate the mRNA and protein expression of peroxisome proliferator‐activated receptor gamma and adiponectin in adipocytes,^26^ which is known to alleviate insulin resistance.^27^, ^28^ LCN2 can also enhance Akt2 phosphorylation at Serine 473 in adipocytes, which is a classical response to insulin stimulation.^26^

In short, with the deterioration of glucose homeostasis and the occurrence of insulin resistance, bone begins to secrete LCN2 into circulation as a compensatory response to suppress food intake and increase energy metabolism. If glucose dysregulation continues to increase, LCN2 acts as an adaptive factor that improves β‐cell proliferation and function to counteract insulin resistance and curbs hyperglycemia.

## 3 LCN2‐MELANOCORTIN‐4 RECEPTOR SIGNALING IN ALL SPECIES

The molecular mechanisms of the appetite suppressing action of LCN2 was subsequently investigated. Intraperitoneal injections of LCN2 in LCN2^−/−^ mice revealed that LCN2 crosses the blood–brain barrier and acts directly in the hypothalamus, a key region involved in appetite regulation.^14^ Interestingly, the suppressive effect of LCN2 on food intake in mice was found to be comparable to that of leptin, another well‐known appetite‐regulating hormone.^29^ Binding of LCN2 to the melanocortin‐4 receptor (MC4R) was identified as a crucial step in mediating its anorexigenic function.^14^ LCN2 was found to colocalize and bind specifically to MC4R in the hypothalamus in mice. This binding was demonstrated to be functional, as LCN2 injection resulted in the upregulation of c‐fos expression in hypothalamic neurons, indicating their activation. Furthermore, using electrophysiological techniques, LCN2 was shown to specifically stimulate MC4R expressing neurons, and following deletion of MC4R, the anorexigenic effect of LCN2 was abolished.^14^

Experiments conducted in nonhuman primates reproduced these observations. To explore the localization and binding of LCN2 in the hypothalamus across species, [124I]rh‐LCN2 was delivered to vervet monkeys and its delivery to the brain was monitored by magnetic resonance imaging and positron emission tomography scans. LCN2 was demonstrated to localize specifically in the hypothalamus, where MC4R is predominantly expressed.^15^ Further, chase experiments using α‐melanocyte‐stimulating hormone as a competitor ligand for MC4R confirmed the specific binding of LCN2 to MC4R in the hypothalamus. This binding pattern was conserved across humans, baboons, and rhesus macaques.^15^

LCN2‐MC4R signaling may also present in humans. In individuals with mutations in the MC4R receptor, LCN2 levels were found to be upregulated.^14^ This upregulation is consistent with a characteristic of G protein‐coupled receptors in which the absence or dysfunction of the receptor leads to an increase in the levels of its ligands. These findings provide initial evidence that the anorexigenic function of LCN2 may be preserved in humans. Indeed, serum LCN2 concentration is inversely correlated with hunger sensation in humans, especially in lean subjects.^15^

## 4 ROLE OF LCN2 IN HEPATIC AND MUSCLE METABOLISM

Nonalcoholic fatty liver disease (NAFLD) is an important metabolic disorder linked with metabolic syndrome, obesity, insulin resistance, type 2 diabetes, and cardiovascular diseases and can progress to simple hepatic steatosis, nonalcoholic steatohepatitis (NASH), and hepatic fibrosis.^30^, ^31^, ^32^, ^33^ Protein and mRNA expression level of LCN2 in the liver were found to be higher in both obese NASH patients^34^ and diet‐induced (high fat/high cholesterol or high fat/high cholesterol/high fructose, HFCF) NAFLD mice.^35^ Hepatic overexpression of LCN2 protects against HFCF‐induced NAFLD by promoting lipolysis and fatty acid oxidation by increasing proliferator‐activated receptor alpha activity.^35^ Moreover, LCN2^−/−^ mice also exhibit heightened susceptibility to hepatic damage consequent to a high‐fructose diet.^36^ Moreover, LCN2 has antiapoptotic cell death effect by mitigating endoplasmic reticulum (ER) stress,^37^ and LCN2 silencing renders liver parenchymal cells more susceptible to ER stress resulting from the activation of nuclear factor‐κB and c‐Jun N‐terminal kinase (JNK) pathways.^38^ This cascade of events is acknowledged as a contributing factor to the development of hepatic steatosis.^39^ These results suggest LCN2 not only as an indicator of metabolic dysregulation but also a mechanism the body use to keep the homeostasis of lipid metabolism in liver.

However, there are other reports demonstrating that LCN2 can promote the progression of hepatic steatosis to NASH by enhancing the cross‐talk between neutrophils and hepatic macrophages^40^ or by activating hepatic stellate cell, a critical signal of leptin‐mediated fibrosis, through matrix metalloproteinase 9 (MMP9)/STAT3 signaling pathway.^41^

Similar to liver, skeletal muscle acts as a reservoir for glucose, playing a crucial role in preventing hyperglycemia and maintaining blood glucose levels within a physiological range. LCN2 protein level was observed to rise after acute high‐intensity exercise or injury.^42^ Following studies revealed that the elevation in LCN2 expression exclusively occurred in activated satellite cells, which are responsible for producing new stem cells and differentiating into myocytes to support muscle regeneration, whereas LCN2‐knockout mice exhibit impaired skeletal muscle regeneration.^43^, ^44^ Although LCN2 gene expression was increased in skeletal muscle of sarcopenic obese mice, whether it is a cause or a result of muscular atrophy in obesity due to inflammation and oxidative stress is not clear.^45^

## 5 THERAPEUTIC POTENTIAL TARGETING ON LCN2

Expanding on the potential therapeutic applications of LCN2, administration of LCN2 in mice leads to appetite suppression, reduced fat mass, and weight loss in both lean and obese mice.^14^ Similar results were found in mice with long‐term exposure to higher levels of LCN2 by genetically overexpressing LCN2.^24^ In nonhuman primates, administration of recombinant human LCN2 also resulted in a significant reduction in appetite and a tendency toward decreased body weight and serum leptin levels in vervet monkeys.^15^ This potentially positions LCN2 as a promising candidate for weight management and metabolic improvement by inhibiting appetite.

As with any medication, assessing its safety profile and potential side effects becomes a critical consideration. The relationship between LCN2 and cancer casts some concern on the safety of LCN2 as a pharmaceutical intervention in the treatment of obesity and other metabolic syndromes. Some studies reported high expression level of LCN2 in some cancer types, such as breast cancer and colorectal cancer, and its expression level was associated with cancer progression, poorer prognosis and decreased overall survival.^46^ The mechanisms of these effects are not clear, possibly by increasing the transportation of iron into cancer cells and inhibiting ferroptosis; interacting with MMP9, a molecule that facilitates the breakdown of the extracellular matrix, thereby promoting the invasion of cancer cells, and several other signaling pathways.^46^

However, the relationship of LCN2 and tumorigenesis still lacks consistent findings, some other reports failed to reveal high LCN2 expression in lymph node metastases,^47^ and elevated levels of LCN2 can also suppress hepatocellular carcinoma cell proliferation and invasion via the interrupting of JNK and PI3K/Akt signaling pathways.^48^ The same results were also reported in pancreatic cancer.^49^, ^50^ Another study showed that overexpression of LCN2 induce tumor apoptosis of colorectal cancer.^51^ It seems to be more a marker than a causal factor. A probable explanation is that LCN2 expression is potently stimulated by inflammation and all cancers have a strong inflammatory component or that it is upregulated as a compensatory mechanism in response to inflammation as an anti‐inflammatory. Thus, more evidence is warranted to elucidate the relationship between LCN2 and cancer.

Although the journey to clinically establish the efficacy and safety of LCN2's anorexic effects is still ongoing, an intriguing perspective emerges: could the development of an anti‐LCN2 agent hold the potential to stimulate appetite, particularly in situations like cancer‐induced cachexia? In fact, a recent study found exceedingly increased LCN2 level in the serum and cerebrospinal fluid of the murine models of pancreatic cancer, which is responsible for suppressed appetite and cachexia. When LCN2 was knockout or its central ligand MC4R was antagonized by agouti‐related peptide, the food intake of these pancreatic cancer mice was restored and the mice did not exhibit cachexia phenotypes.^52^ A more recent study also observed that loss of LCN2 prevents the muscle atrophy in mice with pancreas cancer.^53^ Similarly, the serum concentration of LCN2 in lung cancer patients with cachexia was higher than those without cachexia and was negatively related to BMI and serum albumin levels in these patients. Further experiments revealed that the LCN2 induced ferroptosis in adipose tissue was responsible for tissue wasting in a mice model with lung cancer cachexia.^54^ These results inspire us to see the other side of the coin, that targeting LCN2 by some means like neutralizing antibody may be a promising therapy for cachexia‐anorexia syndrome.^55^ Nevertheless, either LCN2 itself or neutralizing antibody of LCN2 could be a promising drug target, and is worthy of further investigation.

## 6 CONCLUSION AND FUTURE RESEARCH

The discovery of LCN2 as a bone‐derived hormone with anorexigenic effects has provided new insights into the regulation of appetite and glucose homeostasis. Bone‐derived LCN2 can act as a potent suppressor of appetite by crossing the blood–brain barrier, binding to MC4R in the hypothalamus, and activating anorexigenic pathways. It also has insulinotropic effect. The translational potential of LCN2 has been demonstrated in nonobese women and nonhuman primates, highlighting its relevance to human physiology.

However, there are still some research gaps in this field, lots of work are needed in the future, including but not limited in the following aspects:

1. In homeostasis, in a healthy situation, LCN2 is mainly expressed and secreted by osteoblasts. Under inflammatory stress it can be expressed by lung, liver, and kidney. In addition to exerting their endocrine effects upon entering the systemic circulation, hormones secreted by various tissues may also play a local role through paracrine and autocrine mechanisms, leading to distinct functions.^56^ Investigating its tissue‐specific effects, clinical relevance in humans, and therapeutic potential of targeting LCN2 is of interest.
2. A pivotal inquiry revolves around discerning whether elevated LCN2 levels represent compensatory responses or act as direct contributors to the initiation and progression of the diseases. Prospective studies and the application of Mendelian randomization analysis stand as robust methodologies to uncover causal relationships^57^ unraveling the intricate interplay of factors influencing LCN2 dynamics.
3. Addressing the question of how to proficiently manage variables affecting LCN2 becomes paramount, given its heightened presence in many pathological contexts. Strategies to regulate influential factors like immune responses, inflammation, and renal function could provide critical insights into the management of LCN2‐related conditions.
4. Clinical trials evaluating the efficacy and safety of LCN2 in the treatment of obesity and other metabolic syndromes are warranted. The application of anti‐LCN2 molecule^46^, ^58^ to improve anorexia and cachexia in patients with malignancy or anorexia nervosa is another field worthy of further investigations.

## DISCLOSURE

The authors have no conflicts of interest.
